# Higher tactile sensitivity in preterm infants at term-equivalent age: A pilot study

**DOI:** 10.1371/journal.pone.0229270

**Published:** 2020-03-05

**Authors:** Vanessa André, Virginie Durier, Alain Beuchée, Jean-Michel Roué, Alban Lemasson, Martine Hausberger, Jacques Sizun, Séverine Henry

**Affiliations:** 1 Univ Rennes, Normandie Univ, CNRS, EthoS (Éthologie Animale et Humaine)—UMR 6552, Rennes, France; 2 Unité de Soins Intensifs Néonatals, Centre Hospitalier Universitaire de Rennes, Rennes, France; 3 Unité de Soins Intensifs Néonatals, Centre Hospitalier Régional et Universitaire de Brest, Brest, France; University of Bern, SWITZERLAND

## Abstract

Despite a growing body of research on perinatal sensory abilities, data on the extent of tactile sensitivity and more particularly passive touch (*i*.*e*. sensitivity to a stimulation imposed on the skin) are relatively limited, and the development and processing of tactile function are still thus little known. This question is particularly of high importance for infants with atypical early development such as those born prematurely who are exposed to many sensory (including tactile) stimulations (being in a hospital setting) during a critical period of brain development and those born at early term whose birth occurs at the precise time of cortical reorganization, in particular in the sensory areas. Some parents and health-care providers have for instance reported that children born prematurely exhibit atypical (*e*.*g*. higher) sensitivity to “benign” tactile stimuli. In the present study, we hypothesized that preterm and early-term infants may show altered tactile sensitivity. We compared the behavioral responses around term-equivalent age of infants born either pre-term, early-term or at term to the application of a light (0.008 grams) mechanical stimulus. We found that almost all preterm infants perceive this tactile stimulus, contrarily to the two other groups of infants. This extreme tactile sensitivity may be due to experiential, maturational or more likely both processes. We also compared the tactile sensitivity of these infants to that of adults. We found that adults were irresponsive to the light mechanical stimulus. This finding opens not only new insights in understanding development of tactile processing, but also new lines of thought about the particular sensory world of premature and early-term infants and hence about the potential impact of early care practices.

## Introduction

Neonates, and specially premature newborns, had long been considered devoid of sensory perception and even of the ability to feel pain [1]. But, in recent decades, a growing body of evidence, based on numerous studies on the perceptual and cognitive abilities of fetuses and newborns, has demonstrated that sensory systems are relatively mature at (term) birth, at both peripheral and central levels (for reviews: *e*.*g*. [2], [3], [4], [5], [6]). There are different possible explanations for this early undervaluation: one main aspect is that neonates are non-verbal individuals, which means that the initial assessment of their sensory abilities was made by adults caretakers that did not necessarily share the same sensory world; moreover, the immaturity of neonates may have been overestimated, by supposing *a priori* limited sensory and cognitive skills; and at the same time, there were technological limitations that prevented the direct observation of fetal behavior and the study of infant brain function.

Data on perinatal chemosensory and auditory perception are abundant, while the extent of newborns’ tactile sensory capacities has been poorly investigated [6] despite the fact that receptors and neural pathways associated with tactile perception are long known to be the first to develop *in utero* [7]. As soon as the 7^th^ week of gestation, the different types of skin sensory receptors gradually develop to be finally present on the whole skin by the 20^th^ week [8], [9], while the connections between the spinal cord and the brain are functional only by the 20^th^-24^th^ week [10]. Passive tactile stimuli (*e*.*g*. pressures on the maternal abdomen) can already be perceived by the fetus [11], [12], while premature and full-term newborns are known to perceive and react not only to nociceptive stimuli (*e*.*g*. heel lance; *e*.*g*. [13], [14], [15], [16]), but also to lighter stimulations such as abdominal or foot tactile stimulations of only a few grams (*e*.*g*. [17], [18], [19]). Pre-term and full-term infants can also perceive, through touch, objects’ characteristics like shape, texture and weight, thus demonstrating well-developed active touch abilities (*e*.*g*. [20], [21], [22], [23], [24], [25]). The question of the sensitivity to passive touch (*i*.*e*. sensitivity to a stimulation imposed on the individual’s skin) is of particular importance for infants born prematurely (before 37 weeks of gestation) who need intensive medical care and develop in sensory environments (Neonatal Intensive Care Units) dramatically different from those in which full-term infants mature and which consist in numerous sensory (including tactile and sometimes noxious, *e*.*g*. repeated blood sampling) stimulations during a critical period of brain development [26]. In pre-terms, there are discrepancies between studies as some indicate a lower reactivity of premature newborns to tactile stimulations as compared to their full-term counterparts [17], [18], while others point out a higher sensitivity [19], [27], [28], [29], [30] which continues at least throughout the first year [31]. This discrepancy may be due to different factors, especially relative to the location (*e*.*g*. abdomen, thigh, leg, forearm) and/or intensity (*e*.*g*. from 0.6 to 26 g, soft stroke) of the tactile stimulation, as well as the measurements of the subject’s responses. In particular, some authors have examined only the local reflex following a tactile stimulation (*e*.*g*. [19], [27], [28]) while others have taken into account less localized behavioral responses (*e*.*g*. [32]). Differences between premature and full-term infants may exist in terms of specificity and localization of the responses, as demonstrated by EEG and EMG recordings [29], [33]. It has been argued that premature infants may lack the inhibitory circuits or the specialization of required brain areas to respond selectively to relevant tactile stimuli at the appropriate location, showing more extensive responses than full-term infants even for non-nociceptive stimuli [19], [33]. Another category of newborns, the early-term (born between 37 and 38 weeks of gestation), are of interest. Although data are relatively scarce, there is a growing body of evidence that there are risks associated with early-term births such as increased morbidity and lower later cognitive abilities (*e*.*g*. [34], [35], [36], [37], [38], [39]), which has led to the recent reassessment of their “status of full-term” ([34], [40]). Moreover, neural specialization for tactile perception seems to present a critical period of strong brain restructuration around 37 weeks post-conception [33], which makes this precise time of birth of special interest for testing tactile sensitivity.

In the present study, we tested the hypothesis that infants born either pre-term, early-term or at term differ in their tactile sensitivity to passive touch at term-equivalent age. The reasons why they could differ may be differences in terms of maturation and/or sensory experiences (*e*.*g*. exposure to enhanced sensory stimulations in neonatal intensive care units). If the hypothesis that the specificity and selectivity of tactile responses are related to gestational age, we would expect full-term newborns to be less responsive to a light (0.008 g) mechanical stimulus and to have more localized responses. In addition, we hypothesized that adults, who have fully developed inhibitory systems, may not perceive such a light, non-“ecologically-pertinent” stimulation. We therefore compared their tactile sensitivity of that of the infants, using the same experimental device. The results of such investigation are of primary importance as they can give a novel view on how routine practices may be perceived and also on how the newborns’ behaviors may inform us about their perceptions. This work is also important in understanding development of tactile processing in general.

## Methods

### 1) Recruitment

The study took place from November 2015 to July 2016, and was conducted in accordance with the Declaration of Helsinki and approved by the Brest and Rennes Regional and University Hospital Centre ethical committees. The experiment was classified as purely behavioral and the testing involved no discomfort or distress to the infants. The adults and infants’ parents gave written consent to participate (themselves or their babies) in the experiment. They had the option (but were not obliged) to sign an additional document which allows the diffusion of their images or videos for scientific communication. Participant anonymity was retained by identifying each individual by a number.

### 2) Study participants

Twenty-nine infants and thirty young adults were included in the present study.

#### a) Infant participants and groups

Twenty-nine infants, born either preterm (25 to 36 weeks of gestation; N = 10), early-term (37 to 38 weeks of gestation; N = 9) or full-term (39 to 41 weeks of gestation; N = 10) were recruited from the neonatal intensive care units (NICU) and maternity wards of two university hospitals (15 infants from Rennes, 14 infants from Brest) that are known to share the same general practices: on both sites, the NICU followed the developmental care guidelines of the Newborn Individualized Developmental Care and Assessment Program (NIDCAP), which recommend in particular that lights and sounds be reduced as much as possible and the infants‘ activity rhythm be respected. These guidelines encourage parents to visit whenever they want (day or night) and for as long as they wish, and to perform skin-to-skin and routine care when present. To be included in the study, infants had to be without clinical (*i*.*e*. respiratory and cardiac distress) and/or major congenital complications, suspected infections, neurological and sensory perception disorders, and/or under analgesic or sedative treatment.

The characteristics of the three groups of infants are detailed in Table 1:

1. PRE-TERM Group consisted of ten preterm infants (50% females) born at 31.2 (± 3.4) ($\bar{X}$ ± SD) gestational weeks with a mean weight at birth of 1427.5 (± 459.9) g.
2. EARLY-TERM Group was composed of nine early-term newborns (33% females) born at 37.9 (± 0.6) gestational weeks with a mean weight at birth of 2605.6 (± 405.1) g.
3. FULL-TERM group consisted of ten full-term newborns (70% females) born at 40.7 (± 0.5) gestational weeks with a mean weight at birth of 3441.3 (± 451.8) g.

**Table 1:** Characteristics of the populations at birth and at time of testing.

Characteristics	Pre-term group	Early-term group	Full-term group
N (%), mean (SD) or range ([Min-Max])	N = 10	N = 9	N = 10
**Gestational age (weeks)**	31,2 (3,4)	37,9 (0,6)	40,7 (0,5)
**Gestational age [Min-Max] (weeks)**	[26,0–35,1]	[37,0–38,7]	[40,0–41,4]
**Birth weight (g)**	1427,5 (459,9)	2605,6 (405,1)	3441,3 (451,8)
**Birth weight [Min-Max] (g)**	[870–2150]	[2090–3090]	[2780–4078]
**Caesarian delivery**	4 (40%)	4 (44%)	0 (0%)
**Breastfeeding**	7 (70%)	6 (67%)	6 (60%)
**Gender**			
** Girls**	5 (50%)	6 (67%)	7 (70%)
** Boys**	5 (50%)	3 (33%)	3 (30%)
**Chronological age at test (days)**	46,6 (26,7)	3,0 (1,0)	2,0 (0,0)
**Chronological age at test [Min-Max] (days)**	[15–88]	[2–4]	[2–2]
**Post-conceptional age at test (weeks)**	37,8 (0,5)	38,3 (0,6)	41,0 (0,5)
**Post-conceptional age at test [Min-Max] (weeks)**	[37,1–38,6]	[37,3–39,3]	[40,3–41,7]

#### b) Adult participants

Thirty adult participants (50% females; aged from 21 to 36 years old) were recruited. They had all been full-term newborns and did not present any cerebral lesion or known important sensory perception disorders at birth and at the time of the study.

### 3) Testing procedure

The same investigator (VA) performed all the testing and the same equipment for all subjects.

#### a) Testing procedure in infants

Full-term infants were tested 2 days post-birth at the mean post-conceptional age of 41.0 (± 0.5) weeks. Preterm and early-term infants were investigated around term-equivalent age, a few days before discharge. Preterm infants were thus tested at the mean postconceptional age of 37.8 (± 0.5) weeks with a mean postnatal age of 46.6 (± 26.7) days, while early-term infants were tested at the mean post-conceptional age of 38.3 (± 0.6) weeks and at the chronological age of 2–4 days. Testing was conducted in the familiar hospital room in bed during quiet periods. A parent was present during testing, and was asked to remain neutral and to not stimulate the newborn. All infants were lying on their back with their head naturally turned to one side. A test began when the infant was quietly awake and alert, rated in stage 3 on Prechtl’s scale [41] (*i*.*e*. open eyes, no vigorous movements). As neonates spend up to 70% (full-terms) or even 90% (pre-terms) of the time sleeping [42], testing neonates during quiet alert state is particularly time-consuming and demanding, which is probably why previous studies on tactile sensitivity (passive touch) have been performed during active or quiet sleep [17], [18]. However, it is well known that the attentional state of a subject influences his/her responses to sensory stimulations (*e*.*g*. [43]), and as a consequence, testing neonates in a quiet alert state appears more relevant for studies on sensory perception.

Each infant was exposed on the same day to two types of stimulation: one light tactile stimulation and one sham stimulation. Within each group of infants, order of stimulations alternating sham and real varied randomly between subjects (a sham stimulation first: 52% of the subjects; a real stimulation first: 48%; see S1 Table for more details). The light tactile stimulation was conducted with the 1.65 filament selected from Semmes-Weinstein aesthesiometer® which is calibrated to deliver a target force of 0.008 grams (*i*.*e*. the lightest possible) when the filament is slightly bent. During the tactile test, the filament was applied perpendicular to the dorsal surface of the hand (in the center, equidistant from the wrist and bottom of the third finger) until the filament bent slightly as recommended by Andrews & Fitzgerald [28], [44], and each infant was submitted to three consecutive (*i*.*e*. with an inter-stimulus interval of less than 1 second) 1-second subtle tactile stimulations. During the control test (sham stimulation), the experimenter similarly approached three times in rapid succession (overall duration of 3 seconds) the dorsal hand of the infant with the aesthesiometer, but without touching it with the filament (the filament was kept in the retractable protective head). We chose to test the dorsal surface of the hand as this is a part of the body easily accessible and frequently stimulated for care. The experimenter tested the hand opposite to the direction of the infant’s head to prevent her/him from visualizing the device during application and thus ensure behavioral responses were due to tactile stimulation. Although stimulation side was contextually dependent, stimulations were randomly distributed (51.7% for the right hand and 48.3% for the left hand; S1 Table).

#### b) Testing procedure in adults

Adult participants were tested at the Human and Animal Ethology laboratory (EthoS), in a calm and isolated room. They were tested lying on a comfortable deckchair during a calm wakefulness period (*i*.*e*. the participant was just asked to relax) in an isolated room. The testing procedure was similar to that used in infants. The experimenter randomly chose the tested hand. To be sure that the participants did not see the stimulation, they were blindfolded. A second session without blindfold (to limit the tactile stimulations on the face) was performed: the participants were thus asked to keep their eyes closed. As the results did not differ significantly between the two sessions, we further used here only the first session (with blindfold).

#### 4) Data recording and analyses

All sessions were videorecorded using a Sony HDR-PJ350E camera, placed on a tripod facing the participant (at approximately 1 meter from infants and 2 meters from adults). The videos were later analyzed using all occurrences sampling [45]. One experimenter (VA) performed all the behavioral analyses, and another evaluator (SH) blind to the experiment coded again all the videos to test for inter-observer agreement. Comparisons of data between both raters showed high agreement (Cohen’s Kappa: 91%).

#### a) Behavioral analysis in infants

We considered as a reaction to the stimulation (either tactile or sham) any behavioral change (*e*.*g*. arm, leg, finger, head movement) occurring during the second following the stimulation as tactile stimulation of neonates is known to induce reactions after short delays (*i*.*e*. less than 50ms for motor neurons, less than 1s for behavioral reactions), as well as non-localized responses [27], [28], [46]. Emphasis was put only on body movements (*i*.*e*. movement of at least one part of the body) that are known to be better indicators of preterm newborns’ tactile perception than facial expressions [47]. Besides, analyses of facial expressions were not included as the infant’s head was often turned away from the cameras and/or hidden behind a part of her/his body. Results are expressed in absence/presence of a reaction for each test (tactile and sham stimulations). In addition, we rated the behavioral responses (changes in motor activity) on a six-point scale, representing the number of body parts (limbs and head) moved (adapted from [17] and [18]). Ratings ranged from 0 for no limb/head movement to 5, which represented the movement of all four limbs and the head, including startles and gross body movements. Lastly, when infants reacted, we discriminated between localized *versus* non-localized responses. A localized response corresponds to a movement of the touched hand only (with or without a head turn towards the hand), while a non-localized response was a movement of any other body part.

#### b) Behavioral analysis in adults

Behavioral responses to both types of stimulation were recorded following the methodology used in infants. In addition, at the end of an experimental session, the adult participants were asked whether they had perceived anything and, if so, what they had perceived. If no tactile stimulation had been perceived, the experimenter asked them whether they had felt anything on their skin during the session, and if so, at which body location.

### 5) Statistical analyses

As data were not normally distributed, non-parametric statistical tests were used. The statistical tests were performed using Statistica® 13 (Statsoft, Tulsa, USA).

In order to compare whether the proportion of subjects responding changed between the sham and tactile stimulations, McNemar’s tests were conducted within each group of participants. This test is used to compare paired proportions and is applied to a 2 × 2 contingency table, with a dichotomous trait (*e*.*g*. reaction *versus* absence of reaction) and matched pairs of subjects (*e*.*g*. sham *versus* tactile test). Wilcoxon tests were additionally conducted within each participants group in order to compare the intensity of the response to the tactile *versus* sham stimulation. Lastly, Chi-squared tests were used to compare the number of localized (movement of the touched hand only) *versus* non-localized (movement of any other body part) responses.

In order to compare the reactions to both types of stimulation (intensity of the response) scored in the three infant groups (preterm, early-term and full-term infants), Kruskal-Wallis and post-hoc Mann-Whitney tests were used. Fisher’s exact tests were also used to compare the proportion of infants who reacted to the test stimulation to that of adults who reacted and/or reported to have perceived the test stimulation during the experimental session, and to test for potential sex or side effects.

## Results

### Responses of pre-term, early-term and full-term infants to the light tactile *versus* sham stimulation (S1 Table)

**Fig 1:**
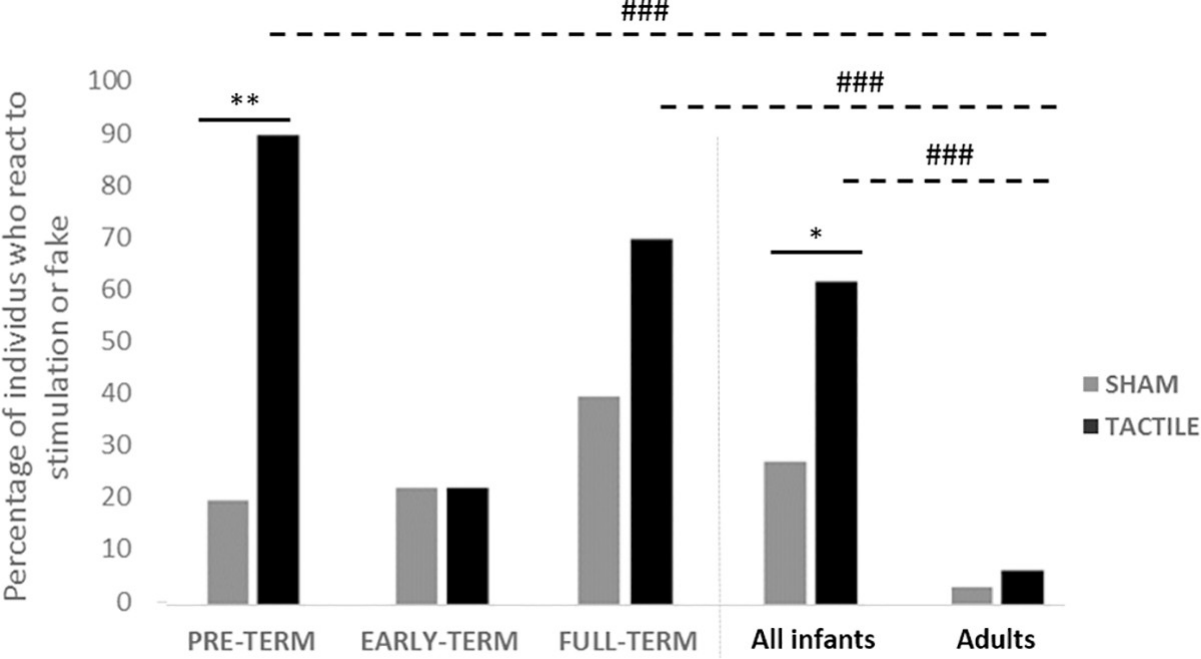
Percentage of preterms, early-terms and full-terms, as well as adults reacting respectively to the tactile and sham stimulations. Statistical analyses: McNemar’s tests: * P<0.05, ** P<0.01; Fisher tests, # # P<0.01; # # # P<0.001.

In total, 18 of the 29 term-aged infants (62.1%) reacted to the tactile stimulation whereas only 8 (27.6%) showed a behavioral change during the sham stimulation (McNemar’s test, N = 29, X^2^ = 5.56, df = 1, P = 0.018) (Table 2, Fig 1). In addition, the mean intensity of the response was higher when exposed to the tactile (X±ES = 1.86±0.36) than the sham stimulation (X±ES = 0.55±0.23; Table 2). The most frequent reaction (14 out of the 18 reacting infants, Chi square Test, N = 18, X^2^ = 4.5, p = 0.034) was a non-localized response, *i*.*e*. a movement of a body part other than the hand touched, to the localized tactile stimulation. No effects of subject’s sex (girls *versus* boys: Fisher Test, N = 29, df = 1, p>0.1) or side of presentation (right *versus* left hand: Fisher Test, N = 29 df = 1, p>0.1) could be evidenced for any of the infant groups.

**Table 2:** Responses of infants (pre-term, early-term and full-term) and adults to both types (tactile *versus* sham) of stimulations.

	Groups of infant participants			Groups of participants	
	**PRE-TERM** N = 10	**EARLY-TERM** N = 9	**FULL-TERM** N = 10	**INFANTS** N = 29	**ADULTS** N = 30
**SHAM STIMULATION**					
Number (%) of participants who reacted *	2/10 (20%)	2/9 (22%)	4/10 (50%)	8/29 (28%)	1/30 (3%)
Mean (± SE) intensity of the response **	0.20 ± 0.13	0.33 ± 0.22	1.10 ± 0.59	0.55 ± 0.23	0.00 ± 0.00
Number of localized responses	1/2	0/2	1/5	2/9	
**TACTILE STIMULATION**					
Number (%) of participants who reacted *	9/10 (90%)	2/9 (22%)	7/10 (70%)	18/29 (62%)	0/30 (0%)
Mean (± SE) intensity of the response **	2.20 ± 0.53	0.33 ± 0.22	2.90 ± 0.71	1.86 ± 0.36	0.00 ± 0.00
Number of localized responses	2/9	1/2	1/7	4/18	
**STATISTICAL ANALYSIS**					
McNemartest (sham *versus* tactile) *	P = 0.008	P = 1	P = 1	P = 0,018	P = 1
Wilcoxon test (sham *versus* tactile) **	P = 0.013	P = 1	P = 0.135	P = 0,007	P = 1

**Fig 2:**
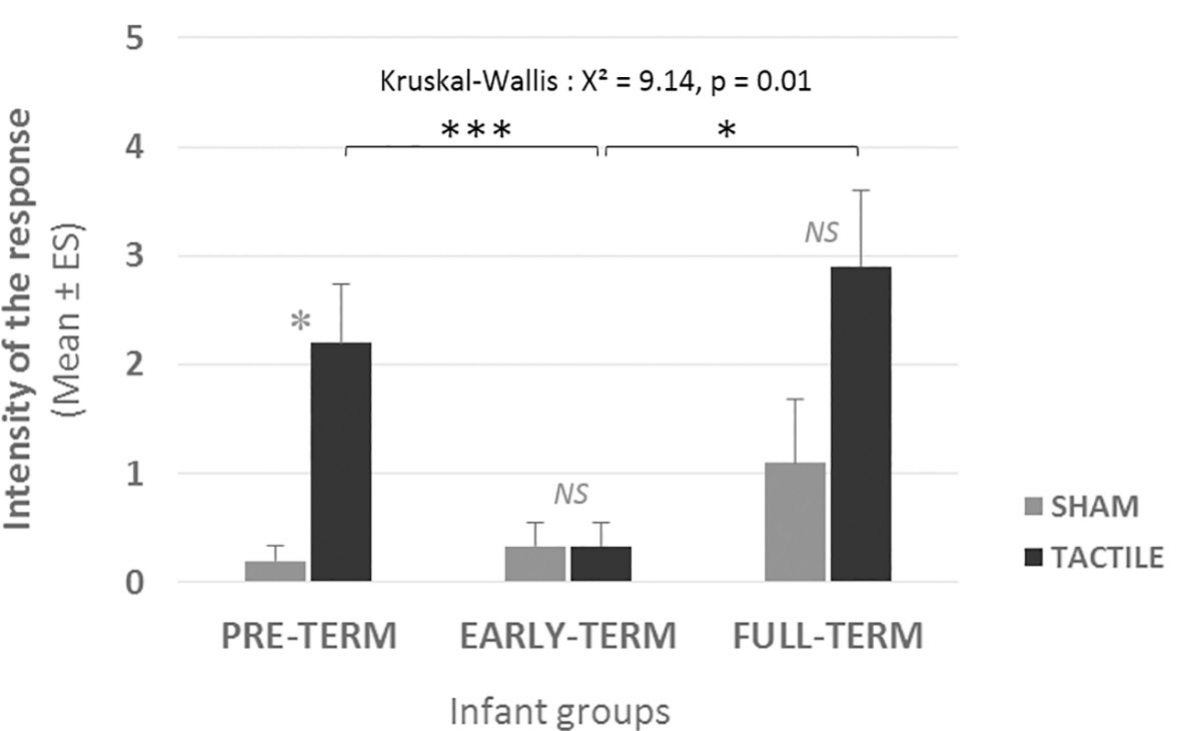
Intensity of the responses of infants (pre-term, early-term and full-term) to both types (sham *versus* tactile) of stimulations. Statistical analyses: Sham *versus* Tactile: Wicoxon test: * p<0.05, NS p>0.1; Comparison of infant groups: Kruskal-Wallis and subsequent Mann-Whitney U-test: * p<0.05, *** p<0.005.

There were clear differences between the three groups of infants as most of the responses to the tactile stimulation were presented by the preterm infants (Table 2, Fig 1). Indeed, almost all pre-term infants (9/10) reacted to the tactile stimulation, whereas only two showed slight behavioral changes during the sham stimulation (McNemar’s test, N = 10, X^2^ = 7, df = 1, p = 0.008). In addition, they reacted more strongly to the tactile stimulation (intensity of response: X±ES = 2.20±0.53) than to the sham one (X±ES = 0.20±0.13; Wilcoxon test: N = 10, p = 0.013) (Table 2, Fig 2). Results were more mitigated in full-term infants as even if seven of the ten full-term newborns reacted to the tactile stimulation, four reacted also to the sham one (N = 10, X^2^ = 1.3, df = 1, P = 1). Early-term infants appeared in overall poorly reactive: the behavior of only two out of the nine early-term infants changed following the tactile stimulation and the sham test (N = 9, X^2^ = 0, df = 1, P = 1). Besides, both early-term (Wilcoxon test: N = 9, p = 1) and full-term (N = 10, p = 0.135) infants did not exhibit a significant increased reaction to the tactile stimulation as compared to the sham one (Table 2, Fig 2).

These differences among the three infant groups were further evidenced when comparing the intensity of reactions to both types of stimulation: while no difference was evidenced between pre-term, early-term and full-term infants for the sham stimulation (Kruskal-Wallis: X^2^ = 1.55, p = 0.46), clear differences were highlighted for the tactile stimulation (X^2^ = 9.14, p = 0.01), with both pre-term and full-term infants showing increased reactions as compared to their early-term counterparts (Fig 2).

### Differences in tactile sensitivity between infant and adult participants

Contrary to infants (18 out of 29), none of the 30 adults showed a behavioral reaction to the tactile stimulation but one reacted to the sham stimulation (Fig 1). Two other adults declared having felt something on their (tested) hand but had expressed no visible behavioral reaction at the time. Thus, two adults may have perceived the stimulation, which is in any case a much lower proportion than for the whole infant group (N = 59, Fisher Test, df = 1, P<0.001), or even the full-term (N = 40, df = 1, P<0.001) and preterm (N = 40, df = 1, P<0.001) infants taken separately (Fig 1). No significant difference with the early-term group could however be evidenced (N = 39, df = 1, P = 0.22; Fig 1).

## Discussion

The results of this pilot study revealed high differences in terms of tactile sensitivity in preterm, early-term and full-term infants at term-equivalent age. Thus, premature infants showed a higher tactile sensitivity as compared to infants born near or at term, confirming earlier reports [19],[30]. Thus, less full-term infants reacted to the very light mechanical stimulation, while almost none of the early-term infants did. This study highlights for the first time that early-term births could be associated also with altered sensory processing despite the fact that those early-term neonates had a weight and Apgar scores at birth in the normal range. In accordance with our hypothesis, adults showed no or almost none evidence of perception of this stimulation.

There are two non-exclusive hypotheses about the processes involved in the differences observed between the groups of newborns:

1) The higher sensitivity observed in the preterm infants could be related to postnatal experiences. At the time of testing, the infants had already spent a long time (from 20 to 88 days) in the neonatal care unit (NICU) where they were submitted to numerous and various procedures, corresponding to a wide range of cutaneous sensory stimuli, either light (*e*.*g*. diaper changes) or strong/noxious (*e*.*g*. venipuncture, intubation) [48], [49], Previous works have in particular highlighted that the degree of reaction to harmful treatments in preterm infants was related to the time spent in hospital after birth and the number of experienced noxious procedures [50], [51]. As tactile stimulation of preterm infants appears to induce sensitization rather than habituation [52], part of our results could be explained by the accumulation of aversive postnatal experiences. It has also been argued that the developing organisms’ sensory system is adapted to the precise stage of their development [53]. Young preterm infants are still at a “fetal” stage when they are supposed to be still stimulated by light tactile stimulations from the womb walls. This could explain why the softer stimulation of kangaroo care (consisting in a skin-to-skin contact between mother and infant which has to start as soon as possible and be maintained as long as possible) can yield better outcomes than a non-“calibrated” massage [54]. The external world, especially neonatal intensive care units (NICU) where preterm newborns are kept, may also over-stimulate their “prenatal” sensory system [55].

2) Preterm infants may also show a differential maturation due to an earlier stage of development of the sensory system at birth. At the skin level, preterm newborns’ epidermal barrier is thinner, more permeable to exogenous materials and more prone to damage than in full term newborns [56] and the density of skin mechanoreceptors maybe higher in preterm newborns (mechanoreceptor density at the 20^th^ week of gestation is known to be greater than in adult [8], [9], [57]). However, central processes are certainly involved too as there are cortical responses to a light tactile stimulation in preterms (aged of 36 gestational weeks) [58] and the volume of grey matter shows a clear linear increase until 40 weeks of gestation [59], [60] with a critical period of strong brain restructuration between 35–37 weeks. According to Fabrizi *et al*. [40], preterm infants, who may have lacked this particular stage of brain maturation, react similarly to nociceptive *versus* non-nociceptive stimulations. Intriguingly, the early-term newborns included in this study differed from the other groups. Their status has been recently revaluated [34], [40] and it is considered that their birth at the precise time of this critical period may explain some of their particularities. Further studies should be performed on a larger sample to confirm the hyporeactivity of early-term newborns, and if so, to understand why they are less sensitive or responsive. The mechanisms underlying such effects are to date largely unknown: only a few hypothesis have been put forward including brain development alterations as mentioned above, physiological immaturity or lower glucose levels [36].

Both maturational and experiential factors are certainly at stake as specialization of neural circuits is also dependent on experience [61]. Further studies, especially longitudinal studies in preterm and early-term infants over the first weeks of life, are required to disentangle the roles of maturation and postnatal experiences respectively. In order to better understand the extent of variations in tactile sensitivity of pre-term and early-term infants in comparison to full-terms, it would also be of high interest to investigate reactions to a larger range of tactile stimulations, by using von Frey filaments of increasing density. More studies of the central and peripheral systems involved in tactile sensitivity would also help understanding the processes. But in any case, the higher sensitivity shown by most preterm and some full-term infants must be taken into account in neonatal care. This characteristic may persist until later stages of development as parents report that children born preterm show a long-term tactile over-sensitivity and reluctance to being touched [62].

Very few adults perceived the 0.008 grams stimulation and the commonly accepted threshold is around 0.06 grams [63]. These findings emphasize the different sensory worlds of neonates and how adults may misinterpret a newborns’ non-localized responses to a localized tactile stimulation. This makes it hard for adult caregivers to assess the newborn’s perception, and in turn make an appropriate response that avoids over-sensitizing the extremely sensitive premature newborn. The supposed lack of inhibitory circuits that allow to restrict responses to pertinent stimuli (*e*.*g*. not aversive) and localization [19], [27] prevents the transformation from an excessive non-organized answer (non-localized or generalized movement to even light stimulations) to a control organized one, regulating and preventing exaggerated answers to non-pertinent stimulations such as this very light stimulus (for a review see: [64]). It is essential for caretakers to be able to recognize such reactions which differ from the usual well known indicators (faces, crying, physiological changes: [33], [49]).

Overall, not much is known about tactile processing in early development and these results contribute to the understanding of the development and processing of tactile function. They open new lines of thought about tactile sensitivity of newborns and may be of significant importance for obstetricians, midwives and child psychiatrists.

## Supporting information

S1 Table (DOCX)
