# Supplementary material for: Higher tactile sensitivity in preterm infants at term-equivalent age: A pilot study
Source: PLoS One. 2020 Mar 5;15(3):e0229270. doi: 10.1371/journal.pone.0229270 (PMC7058305; doi:10.1371/journal.pone.0229270)
Supplement: S1 Table — (DOCX) [file pone.0229270.s001.docx]

**S1 Table. Responses of preterms, early-terms and full-terms to both types (sham *versus* tactile) of stimulations.**
